# Supplementary material for: The interactome of intact mitochondria by cross-linking mass spectrometry provides evidence for coexisting respiratory supercomplexes
Source: Mol Cell Proteomics. 2017 Dec 8;17(2):216–32. doi: 10.1074/mcp.RA117.000470 (PMC5795388; doi:10.1074/mcp.RA117.000470)
Supplement: Supplemental Data [file supp_RA117.000470_133922_0_supp_23978_fzffwf.zip › spectra_annotation/mito_DR_spectra_annotation/181-1-3-1-10-1.pdf]

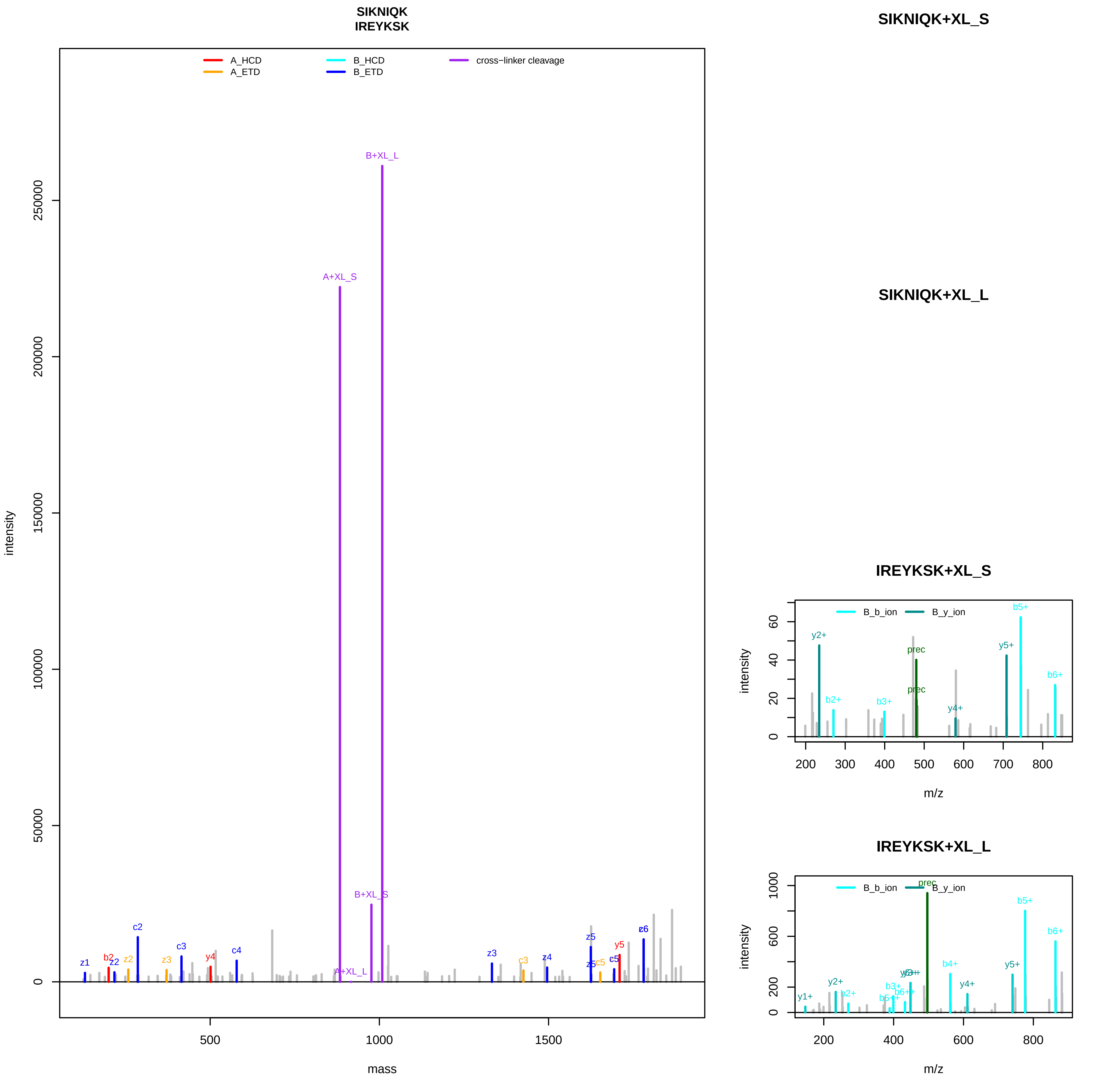
SIKNIQK
IREYKSK
A_HCD
A_ETD
B_HCD
B_ETD
cross−linker cleavage
B+XL_L
A+XL_S
B+XL_S
A+XL_L
z1
b2
z2
z2
c2
z3
c3
y4
c4
z3
c3
z4
z5
z5
c5
c5
y5
z6
c6
intensity
mass
0
50000
100000
150000
200000
250000
500
1000
1500
SIKNIQK+XL_S
SIKNIQK+XL_L
IREYKSK+XL_S
B_b_ion
B_y_ion
y2+
b2+
b3+
prec
prec
y4+
y5+
b5+
b6+
intensity
m/z
IREYKSK+XL_L
y1+
y2+
b2+
b3+
b5++
b6++
y3+
b4+
y4+
y5+
b5+
b6+
intensity
m/z
